# Alterations in volumes and MRI features of amygdala in Chinese autistic preschoolers associated with social and behavioral deficits

**DOI:** 10.1007/s11682-018-9853-9

**Published:** 2018-02-26

**Authors:** Zongming Zhu, Xiangming Fang, Hongwei Chen, Xiangwen Zhu, Lili Zhang, Xiaodong Zhai, Zhimin Cui, Quansheng Gao

**Affiliations:** 1 Department of Radiology, Wuxi People’s Hospital, Nanjing Medical University, No. 299 Qingyang Road, Nanchang District Wuxi, 214023 China; 2 Department of Child Care, Wuxi Children’s Hospital, Nanjing Medical University, Wuxi, 214023 China; 3 Laboratory of the Animal Center, Academy of Military Medical Sciences, No. 27 Taiping Road, Haidian District Beijing, 100850 China

**Keywords:** Preschool children, Amygdala, 3.0T MRI, Childhood autism, Pervasive developmental disorder

## Abstract

To examine the amygdala volume in 2–5-year-old preschool children with autism and explore the relationship between amygdala volumes based on MRI findings and clinical features. A total of 39 cases with clinically diagnosed autism were collected. The oblique coronal T1 weighted image (T1WI) sequence was used to measure the volume of amygdala and the MRI signals were measured and analyzed. The data were compared to that of 24 age-matched healthy children and correlated to the clinical manifestations. The autism and the control groups were subject to brain scanning in 1 week after Diagnostic and Statistical Manual of Mental Disorders, Fourth Edition (DSM-IV) review. The 39 cases, diagnosed with autism, were associated with social and behavioral deficits through clinical observation, physical and neurological examination, and assessments according to DSM IV, and the range of ABC scores in the autism group was 47–124, with an average score of 84.7 ± 24.1. Abnormal MRI signals were found in 19/78 (24.4%) amygdala in the autism group, the amygdala lesions showed punctuate or flaky low signal, slightly low signal, low to iso-signal, slightly high signal, or iso to high-signal intensity on T1 weighted three-dimendional fast low angle shot(T1FL3D) images. The right amygdala volume average was 1.088 ± 0.38 cm^3^, while that of the left amygdala was 1.04 ± 0.41 cm^3^, without any statistically significant difference (t = 0.533, p = 0.596) in the autism group. Among the 24 cases in the control group, the right amygdala volume average was 0.754 ± 0.194 cm^3^, while that of the left amygdala was 0.666 ± 0.252 cm^3^; the autism group had a significantly larger right and left amygdala volumes as compared to the age-matched typically developing group with a significant positive correlation between age and right amygdala volume (r = 0.406, p = 0.01). The preschool children with autism had significantly larger bilateral amygdala volumes as compared to age-matched typically developing children, the amygdala lesions may show abnormal signal. A relationship between age and right amygdala volume in the preschool children with autism was established.

## Introduction

Childhood autism is a representative disease of pervasive developmental disorder (PDD). The incidence of autism in children is approximately 2.38% in China (Li et al. 2011). Childhood autism is prevalent in males, involving a broad range of mental, behavioral, and psychological disorders, such as verbal and cognitive deficits and impairment of social ability. Clinically, this disease displays an early onset in infantile stage accompanied by characteristic manifestations of delayed development in language and communication ability and stereotypical patterns of behavior (Rapin and Katzman 1998). Childhood autism may impose a devastating and a long-lasting impact on several aspects of the lives of patients and their families. In severe cases, the symptoms of childhood autism last throughout the life, and many autistic children cannot survive independently even after reaching adulthood. Thus, the early diagnosis of childhood autism is critical for early and effective therapy.

Courchesne et al. and Sparks et al. (Courchesne et al. 2001, 2002), through brain structure imaging, found that the brain volume of 2–4-year-old autistic children is larger than the normal control group. Billeci et al. (2012) detected that the abnormal white matter integrity might be associated with the pathophysiological process of the autism spectrum disorder. Nagy et al. (2003) showed that the fractional anisotropy(FA) values is reduced in brain regions including prefrontal ventromedial, anterior cingulate, temporoparietal cortex, bilateral superior temporal gyri, bilateral temporal lobes adjacent to the amygdala, and occipital temporal cortex; the abnormalities in these regions may damage the social cognitive function in autism. Weinstein et al. (2011) demonstrated that children with autism had increased FA values in the corpus callosum and left cingulate, and these areas are closely related to the social, emotional, and communication barrier.

The amygdala plays a major role in the maintenance of normal emotional and behavioral pattern as well as the development of the cognitive ability (Adolphs 2008). The emotional, behavioral, and social impairments observed in childhood autism may be associated with the structural and functional abnormalities of amygdala (Werner et al. 2000). Reportedly, the aberrant emotion and behavior in childhood autism are associated with the volume of amygdala (Munson et al. 2002, 2006; Stigler et al. 2011). Moreover, the core behavioral characteristics of the autism diagnosis in children also indicate the dysfunction of the amygdala (Nordahl et al. 2012). Therefore, the measurement of the volume of amygdala by MRI and analysis of the features on MRI signals may provide a novel approach for the early diagnosis of childhood autism, which is the key for early application of behavioral interference and individualized therapy.

The 3.0T MRI technique, with its advantages of non-invasiveness, high spatial resolution, and improved contrast for soft tissues, has been widely applied for studying the brain diseases. High-field MRI has provided superior sensitivity and accuracy over other imaging techniques with respect to the characteristics of signals and boundaries of the amygdala.

In preschool children undergoing rapid development of linguistic ability, the growth of left or right amygdala does not appear to correlate directly with age (Ortiz-Mantilla et al. 2010). A large amount of preliminary data concerning childhood autism and amygdala were collected in Western countries. However, limited data are available on Asian population, and no relevant study has been carried out on Chinese childhood autism. Thus, the present study aimed to analyze the volume and features of MRI signals of amygdala associated with social and behavioral deficits in Chinese preschool autistic patients in order to build a foundation for large-scale clinical studies. Such ethnicity-specific information might be beneficial for the evaluation and early diagnosis of autism in Chinese population.

## Materials and methods

### Subjects

This study fulfilled the criteria of the Declaration of Helsinki. The study protocol was approved by the Research Ethics Committee of the Wuxi People’s Hospital Affiliated to Nanjing Medical University (Jiangsu province, China). Written consent was obtained from the children’s legal guardians before data collection.

A total of 39 patients from February 2013–July 2015, at the Department of Child Hygiene of Wuxi Children’s Hospital, were diagnosed with autism associated with social and behavioral deficits through clinical observation, physical and neurological examination, and assessments according to the “Diagnostic and Statistical Manual of Mental Disorders (DSM IV)” (American Psychiatric Association 1994). The parents of the patients filled the Autism Behavior Check (ABC) List (Krug et al. 1980, 1993), using ABC > 67 as the cutoff value. The ABC scores between 30 and 66 were considered as suspected cases, and their diagnosis was ascertained by considering the clinical manifestations and results of neural examination. The continuous recruitment of the patients was based on the following exclusion criteria: (1) age beyond the 2–5-year range; (2) history of craniocerebral trauma; (3) neural disorders, Rett’s syndrome, childhood disintegrative disorder, selective mutism, obsessive–compulsive disorder, and Asperger’s syndrome; (4) idiopathic language retardation; (5) history of severe somatic disease.

A total of 24 healthy children (18 boys, 6 girls; median age 3 years), included in the control group, underwent medical examination. The individuals of the control group were age-matched and recruited to the autism group through advertisement. The inclusion criteria were as follows: Han ethnicity; healthy, no history of craniocerebral trauma or intracranial disease; no history of epilepsy or psychiatric disease; no somatic disease; no history of seizure. Parents of children filled the same DSM form. The control group consisted of children with intellectual quotient (IQ) > 70, and the subjects and their families did not present any history of neurological or mental illness. None of the individuals from both groups received drug therapy for psychiatric diseases.

### MRI examination

All subjects underwent imaging on a 3.0T MR scanner-imager (Siemens MagnetomTrio Tim, Erlangen, Germany). MRI was performed on autistic patients after the first week of clinical diagnosis at the Department of Medical Imaging of Wuxi Hospital Affiliated to Nanjing Medical University. Children in the control group underwent MRI examination during the first week of recruitment. All MR examinations were conducted at noon or 8:00 p.m. (sleep time) after 10% chloral hydrate was administered orally, allowing 30 min for sedation and hypnotism. The vital signs were monitored during the MRI procedure in T1WI, T2 weighted image (T2WI), FLAIR (fluid attenuated inversion recovery), and T1FL3D sequences. The images were collected on 3.0 MRI scanner using a standard head coil and the following parameters: T1WI (TR = 200 ms, TE = 2.46 ms), T2WI (TR = 4000, TE = 93 ms), FLAIR (TR = 9000 ms, TE = 93 ms), T1FL3D (TR = 19 ms, TE = 4.92 ms), field of view = 23–25 cm, matrix size = 320 × 320, slice thickness = 1.0 mm, sagittal orientation, total scanning time = 10.3 min.

These images were transferred to Syngo via 102230 workstation and opened using MM 3D. VOI Freehand was used for the measurement of volume at 1 mm thickness in the T1FL3D images according to the boundaries of amygdala drawn manually on the image.

On this workstation, the amygdala anatomical boundary was illustrated manually on the coronary images with 1 mm thickness. Subsequently, the volume of amygdala was measured by VOI Freehand volume measurement tool, artificial depiction based on the guidelines for amygdala tracking, and the guidelines of the anatomical structures of amygdala based on the differences in gray and white matter.

### Imaging and analysis of amygdala boundaries

**Fig. 1:**
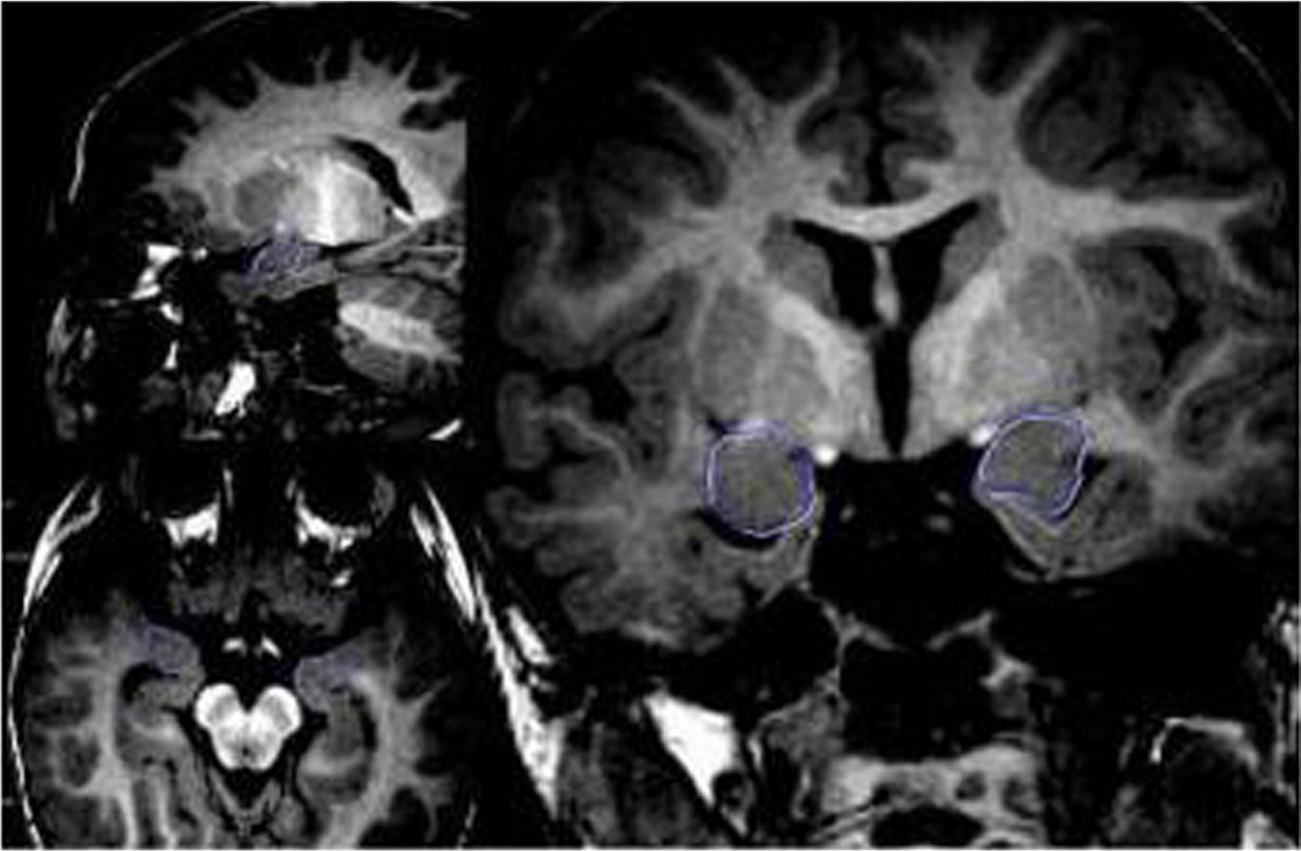
Sagittal, axial, and coronal view of T1WI on MRI sections. A three-dimensional reconstruction of images showed bilateral amygdala with the signal of gray matter and the reference lines for measuring the volume (blue circles)

As shown in Fig. 1, the amygdala boundary was determined based on the previously published methods (Dziobek et al. 2010). The boundaries of amygdala were drawn by two radiologists (one Associate Chief Physician and one Chief Physician) in a double-blind manner (the subjects and the researchers were blinded to the autism or control group); each radiologist had > 10 years of experience in neuroradiology. The discrepancies in the results were discussed between the radiologists to achieve a consensus. However, in the case of continued disagreement, the data were not entered for analysis.

### Data analysis

SPSS 13.0 software was used for the statistical analysis of the data. T-test was used to determine the normal distribution of the quantitative data. The non-normal distribution of quantitative data was converted to meet the normal distribution, followed by t-test analysis. All quantitative data were tested as normal distribution and represented as mean ± standard deviation (SD). The amygdala volume and ABC scores between the autism group and the control group were compared using the Student’s t-test. The gender distribution between the two groups was compared using the Chi square test. Pearson’s correlation analyzed the relationship between the volumes of both sides, left or right amygdala *vs*. age or ABC scores. p < 0.05 was considered as statistically significant.

## Results

### Comparison of ABC scores

The 39 autism cases belonged to Han ethnicity, which represents the majority of the Chinese population. The cohort comprised of 35 (89.7%) males and 4 (10.3%) females, within an age range of 2–5 (median age, 3) years. All the cases exhibited different extents of stereotypical behaviors, as well as, social and behavioral deficits. Other clinical manifestations included linguistic retardation (36 cases) or impaired physical development (mental retardation > 12 months than children with the same age). 37/39 cases displayed a deficit in the IQ.

The range of ABC scores in the autism group was 47–124, with an average score of 84.7 ± 24.1. Only 1 individual had an ABC score < 67 (ABC score47). This individual was later ascertained to be autistic after a comprehensive assessment of the clinical findings. The range of ABC scores in the control group was 3–27 (average, 14.54 ± 7.35). The demographic information and ABC scores are summarized in Table 1.

**Table 1:** Baseline data, ABC total score, ABC average score of the two groups

	Autism group (n = 39)	Control group (n = 24)	Statistical values	*p*-value
Gender (M/F)	35/4	18/6	χ^2^ = 3.571	0.059
Age (years)	2.15 ± 0.70	3.64 ± 1.08	t=-0.949	0.347
ABC scores range (median)	47–124 (97)	3–27 (12)		
Average ABC scores	84.67 ± 24.14	14.54 ± 7.35	*t* = 16.914	p < 0.001

### Morphological comparison of amygdala in the two groups

The transverse diameter of the amygdala in the control group (maximum diameter perpendicular to the median sagittal plane), greater than the anteroposterior diameter (maximum diameter parallel to the lowest point of the temporal pole of the brain and the lowest point of the occipital pole), was almond-shaped or olive-shaped. In the autism group, the transverse diameter of amygdala in 31 cases (79.5%) was larger than the anteroposterior diameter. The morphology of amygdala in the autism and control groups is shown in Table 2.

**Table 2:** Morphological comparison of amygdala in the two groups

Autism group	Autism group (n = 39)	Control group (n = 24)
Olive-shaped (n)	18	20
Almond-shaped (n)	13	4
Cashew type (n)	6	0
Oblong-shaped (n)	1	0
Polygonal-shaped (n)	1	0

In 37 cases, the boundaries of amygdala could be defined clearly. In 2 cases, the boundaries of the amygdala were unclear (one on the left amygdala and the other on the right side). Subsequently, 2 cases were defined as the upper boundary of amygdala, presenting the almond nucleus, while the crustacean gray matter of the thin layer of white matter fibers was unclear. Thus, the boundaries needed to be distinct in order to allow the loss of amygdaloid nucleus and a small part of the central nucleus; however, neither the shell nor putamen and claustrum were included in the amygdala range.

### MRI signal of comparison of amygdala between the two groups

**Fig. 2:**
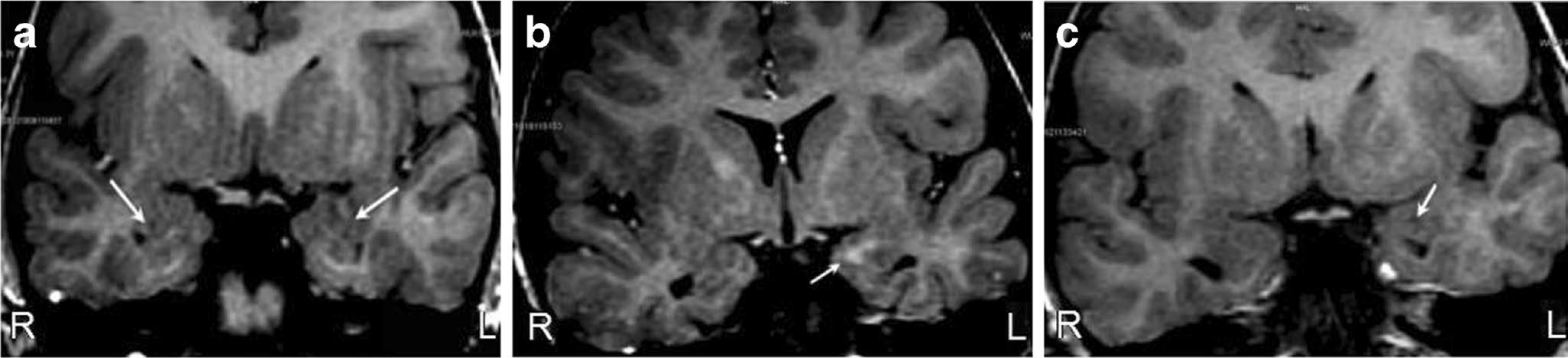
**a** The images of a 2-year-old boy from the autism group. Fl3D T1WI at the oblique coronary position, which shows a nearly-circle flake low signal in the right amygdala (white arrow) along with the mixed signal within the left amygdala (white arrow). **b** The images are of a 3-year-old boy from the autism group. Oblique coronary T1FL3D. A striped high signal in the left amygdala (white arrow). **c** The images of a 4-year-old boy from the autism group. Coronary Fl3D T1WI shows a nearly-circle flake low signal in the left amygdala (white arrow)

Abnormal signals were found in 19/78 (24.4%) amygdala in the autism group (the signal of the amygdala in the control group served as the reference). One patient exhibited abnormal signals in bilateral amygdala (Fig. 2a), and 4 patients showed abnormal signals on the right amygdala. Of these 4 patients, 1 showed punctate low signal in the center of amygdala, while the other 3 displayed spotted or flake-like low signal in the same location (Fig. 2a). Thirteen patients presented abnormal signal in the left amygdala, among whom, 1 showed small flake-like high signal (Fig. 2b), 4 displayed low to iso- signal, 4 showed punctate or small flake-like low signal (Fig. 2c), 2 exhibited low/high mixed signal, and 2 showed iso-high mixed signal. Compared to the autism group, none of the control subjects displayed abnormal MR signals.

### Comparison of the volumes of amygdala in both groups

As shown in Table 3, the volumes of right amygdala in 39 autism children ranged from 0.45 to 2.0 (average, 1.088 ± 0.38) cm^3^, while that of the left were distributed from 0.37 to 2.06 (average, 1.04 ± 0.41) cm^3^. No statistically significant difference was found between the two sides (t = 0.533, p = 0.596). In the control group, the volumes of the right amygdala were 0.34–1.17 (average, 0.754 ± 0.194) cm^3^, while that of the left were 0.34–0.92 (average, 0.666 ± 0.252) cm^3^. Importantly, the total volumes of the amygdala in the autism group were significantly higher than those in the control group (t = 5.901, p < 0.001). Similarly, the volumes of left (t = 5.390, p < 0.001) and right (t = 4.628, p < 0.001) amygdala were significantly higher than those in the control group.

**Table 3:** Volumes of amygdala in autism and control groups (cm^3^)

	Total volume (R + L)	Left	Right	*p*-value
Autism (n = 39)	2.137 ± 0.371	1.088 ± 0.38	1.04 ± 0.41	0.596
Control (n = 24)	1.391 ± 0.687	0.54 ± 0.194	0.666 ± 0.252	0.184
Statistical values	t = 5.901	t = 4.628	t = 5.390	
*p*	< 0.001	< 0.001	< 0.001	

### Correlation between the volumes of amygdala and age or the ABC total score and five factors in both groups

As shown in Tables 4 and 5. We found the total volumes of the amygdala and the volumes of right or left amygdala were not significantly correlated to the age in the control group. In the autism group, the total volumes of the amygdala and the volumes of right or left amygdala were not significantly correlated to the age. In the autism group, the total volumes of the amygdala and the volumes of right or left amygdala were not significantly correlated to the ABC scores. In the autism group, the total volumes of the amygdala and the volumes of right or left amygdala were not significantly correlated with the ABC total score and factors 1–5 (sensory stimuli, relation, body and object use, language, and social self-help) scores.

**Table 4:** Correlation between volumes of amygdala and age or ABC scores of the autism group

Amygdala volumes by MRI	Clinical features	r	*p*-value
Right amygdala volume	Age ABC Scores	0.232 0.147	0.155 0.372
Left amygdala volume	Age	− 0.003	0.986
	ABC Scores	− 0.012	0.941
Total amygdala volume	Age	0.264	0.105
	ABC Scores	0.086	0.601

**Table 5:** Correlation between volumes of amygdala and scored of ABC five dimensions of the autism group

Amygdala volumes by MRI	ABC1-5 scores	r	*p*-value
Right amygdala volume	ABC-1 (Sensory stimuli) ABC-2 (Relation) ABC-3 (Body and object use) ABC-4 (Language) ABC-5 (Social self-help)	0.134 0.201 -0.007 0.129 0.262	0.414 0.220 0.964 0.434 0.107
Left amygdala volume	ABC-1 (Sensory stimuli)	− 0.024	0.884
	ABC-2 (Relation) ABC-3 (Body and object use) ABC-4 (Language) ABC-5 (Social self-help)	0.174 − 0.141 − 0.023 0.186	0.289 0.392 0.887 0.257
Total amygdala volume	ABC-1 (Sensory stimuli)	0.067	0.684
	ABC-2 (Relation) ABC-3 (Body and object use) ABC-4 (Language) ABC-5 (Social self-help)	0.263 − 0.039 0.089 0.272	0.106 0.813 0.590 0.094

## Discussion

The present study provides evidence that Chinese autistic preschoolers were associated with social and behavioral deficits, presenting an abnormal signal in the amygdala. The volume of bilateral amygdala is larger than that of age-matched normal children (2–5 years). Growth, children with right amygdala volume can be increased. Although the ABC score reflects the severity of autistic behavior in children, no significant correlation between the amygdala volume and ABC total score was established. Factors 1–5(sensory stimuli, relation, body and object use, language, and social self-help) scores led to the speculation that although the amygdala volume of children with autism increased significantly, no evidence was found that the degree of increase was related to the degree of clinical disease.

MRI is a non-invasive and low-risk procedure capable of delivering high resolution and tissue contrast. It is a relatively accurate and easy-to-standardize technique, especially suitable for studying childhood autism and juvenile brain structure alterations (Szabo et al. 2001; Eliez and Reiss 2000). In addition, MRI has been used to measure the aberrant changes in amygdala; specific morphological deviations have been detected in childhood autism.

A close association between the enlargement of the amygdala and impaired socialization has been reported in 3-4-year-old autistic patients (Munson et al. 2002, 2006). The severity of the impairment in social integration was found to be quantitatively correlated to the increased size of amygdala (Adolphs 2008). Several studies documented a 15–22% increase in the volume of amygdala in autistic patients < 10-year-old (Munson et al. 2002; Hazlett et al. 2006; Mosconi et al. 2009). Kim et al. reported a 11.0–12.7% increase in the amygdala volume in 6-7-year-old autistic children as compared to the age-matched normal children (Kim et al. 2010). In the current study, the morphological measurement of amygdala volume by MRI revealed that the average volume in autistic patients was 40% more than that in the age-matched normal children. On the other hand, in comparison to those age-matched subjects in Western countries, reduced amygdala volumes are observed in Chinese preschool patients with unilateral and bilateral autism. This phenomenon might be attributed to the racial differences since Asian individuals do not have the same physique as their Western counterparts. This might also explain the reason why Chinese preschoolers have substantially enlarged amygdala volumes as compared to the age-matched subjects as described in other studies. Moreover, in contrast to the data reported previously by Juranek et al. (2006), we did not observe a significant correlation between the increased volume of amygdala and age. These discrepancies could be partially attributed to the relatively large deviations in the head circumferences and differences in the total brain volume (Courchesne et al. 2003; Dawson et al. 2007; Dementieva et al. 2005; Hazlett et al. 2005) among the 2–6-year-old children; a smaller sample size could be another factor. Unlike other organs, the size of amygdala, which is a part of the brain system, will not increase continually along with the growth of children. However, whether the amygdala enlarges further or reverts to normal size or shrinks when autistic children reach the juvenile stage is yet controversial.

While MRI technique has been utilized several times to measure the changes in the amygdala volume in autism patients, only limited data is published on the signal characteristics of this disease. Adolphs et al. (2005) reported that MRI could detect the lesions in both sides of the amygdala in patients with cognitive deficits and apatheia; however, it did not describe the characteristic changes in MRI signals. The current study found abnormal signals on both sides of the amygdala (4 in the right side, 13 in the left side, and 1 bilaterally). The MRI in autistic patients displayed homogeneous low or high signal. Although this is not a highly specific characteristic, it may be used as circumstantial evidence for the radiological diagnosis. In addition, monitoring the changes in the signal character could provide an approach for the evaluation of progression during the return visit of patients.

ABC scores have been applied for the assessment of the severity and behavioral changes in childhood autism or as a screening tool in preschoolers. These scores comprehensively indicate the degree of impairment in patient behavior. Interestingly, the current study did not reveal a quantitative correlation of the score of ABC and five factors with the increase in the amygdala volume. This result suggested a complex nature and diversity in the function of amygdala and clinical manifestations in childhood autism that are not directly determined by the physical changes in the tissue.

Nevertheless, MRI was employed in the present study to measure the size of amygdala and evaluate the signal characteristics as the research focus to identify the differences between preschoolers with autism and healthy children and guide the early clinical intervention in children with autism. The MRI scan displayed a small portion of the frontal and occipital lobes in the cranial MRI image that was not included in the T1FL3D scan, and therefore, the whole brain volume measurement could not be performed. This is also the research direction of our future study. Owing to the limited sample size of female patients in this study, we could not determine a gender-specific difference in the volume alterations among the autistic patients. In addition, the present study was performed on a population representing the Chinese Han ethnicity, and thus, the results may represent the specific characteristics of this population. Hence, future study with a large sample size and simultaneous examination of the function of the amygdala may enable a comprehensive analysis of the pathological mechanisms underlying childhood autism.

In conclusion, we measured the volumes of the amygdala with MRI and found a significant increase in patients with childhood autism as compared to the age-matched controls in a Chinese Han population. No direct correlation was established between the amygdala volume, ABC score and five factors or age in these patients. A large number of homogeneous image signals were observed in the amygdala in the autism group than the control group. This study indicated that MRI could be applied to measure the morphological changes of amygdala in autism patients, and such procedure may be useful for the assessment and diagnosis of childhood autism.
